# Identification of stable internal reference genes for expression analysis in the liver and pancreas of diabetic mouse (*Mus musculus* L.) models under physiological, pathological and treatment conditions

**DOI:** 10.1371/journal.pone.0338403

**Published:** 2026-01-07

**Authors:** Van Diep Nong, Duc Quan Nguyen, Thi Trang Do, Thi Huong Giang Tran, Thanh Hien Nguyen, Tat Thanh Le, Huy Thinh Tran, Huy Hoang Nguyen

**Affiliations:** 1 Hanoi Medical University, Kim Lien, Hanoi, Vietnam; 2 Bac Kan General Hospital, Thai Nguyen, Vietnam; 3 Institute of Biology, Vietnam Academy of Science and Technology, Nghia Do, Hanoi, Vietnam; 4 Publishing House for Science and Technology, Vietnam Academy of Science and Technology, Nghia Do, Hanoi, Vietnam; 5 Graduate University of Science and Technology, Vietnam Academy of Science and Technology, Nghia Do, Hanoi, Vietnam; University of South Africa - Florida Campus: University of South Africa - Science Campus, SOUTH AFRICA

## Abstract

Accurate gene expression analysis via reverse transcription quantitative real-time PCR is vital for studying diabetes mellitus. Accurate and reliable normalization of RT-qPCR data, in accordance with the MIQE guidelines, demands a group of at least two stably expressed reference genes, especially in diabetic models where metabolic dysregulation often alters the profile of gene expression. To identify the best reference genes, this study systematically evaluates the stability of eleven candidate reference genes, including *ACT*, *B2M*, *GAPDH*, *HPRT1*, *PPIA*, *RPL13A*, *RPLP0*, *TBP*, *UBC*, *YWHAZ,* and *18SRNA*, in liver, pancreas, and both types of tissues across four mouse groups of control, obesity, STZ-induced diabetic and treated STZ-diabetic groups. The identification of a robust group of reference genes was conducted by four widely used statistical algorithms of geNorm, NormFinder, BestKeeper, and comparative ΔCt methods. Each applies different statistical algorithms: geNorm uses pairwise variation, NormFinder considers inter- and intra-group variance, BestKeeper uses Ct variability through standard deviation and correlation analysis, and the ΔCt method evaluates expression consistency by evaluating the geometric mean of the stability rankings of each gene across all methods. Our analysis identified *ACT* and *RPL13A* as the most appropriate reference genes for liver, while *UBC* and *RPL13A* were most stable in pancreas. Overall, *RPL13A* and *UBC* demonstrated the highest expression stability across both liver and pancreatic tissues. Furthermore, our findings laid foundations for future studies in the analysis of gene expression in obese, diabetic, and diet-changed mice, as well as other liver- and pancreas-related diseases in mouse models.

## Introduction

Diabetes mellitus, a chronic metabolic disorder characterized by hyperglycemia, insulin resistance, and impaired *β*-cell function, is becoming increasingly prevalent as a result of various factors, including obesity, genetic disorder, and unhealthy lifestyles. Diabetic conditions profoundly impact metabolically active organs, especially the liver and pancreas, leading to disturbances in glucose metabolism, inflammation, and oxidative stress [1]. The condition of diabetes is associated with a range of complications stemming from elevated blood sugar levels, such as cardiovascular diseases, eye injury, kidney disease, and nerve damage, etc. [2,3].

For studying diseases in humans, several animal models have been employed. Among them, the mouse model is extensively used in research due to its remarkable genetic (99%) and physiological similarities to humans [4]. Research indicates that diets containing a high content of saturated fats (lard, butter, ghee, coconut oil, palm oil, etc.) are associated with increased body weight and pre-diabetic conditions in various animal models, including several strains of mice (the NOD strain for T1DM and the BKS, db/db, KK-Ay, and FVB/NJ strains for T2DM) [5]. A diabetic mouse model, developed by a high-fat diet (HFD) to induce obesity and insulin resistance, then followed by a low dose of streptozotocin (STZ) injection, is one of the most popularly used models for investigating the molecular mechanisms underlying T2DM diabetes [2,6]. STZ is a glucosamine–nitrosourea antibiotic, which is primarily used in experimental research to induce diabetes mellitus in animal models, including mice [7,8]. Its diabetogenic effect is coming from the structural similarity to glucose and the selective toxicity to pancreatic β-cells. After entering β-cells through the glucose transporter 2 (GLUT2) route, STZ acts as an alkylating agent that triggers β-cell necrosis and disrupts the production of insulin, therefore leading to the development of diabetic symptoms (insulin insufficiency and insulin resistance) [7,8]. In certain experimental settings, bioactive compounds are utilized as biological modulators to investigate molecular responses under specific treatment conditions [9–11]. Among these, curcumin, a natural polyphenolic compound sourced from *Curcuma longa* L., has been widely recognized as a bioactive compound for its ability to modulate glucose metabolism, hepatic gluconeogenesis, *β*-cell activity, insulin signaling, insulin sensitivity, oxidative stress and inflammation in liver and pancreatic tissues [12–16]. Therefore, curcumin was included in this study as a biological modulator in diabetes-related research.

Quantification of gene expression in target tissues is crucial for understanding the complex signaling networks and pathological responses as well as treatment effects in various studies, all of which primarily employ reverse transcription quantitative real-time polymerase chain reaction (RT-qPCR) [17,18]. In diabetic mouse models, the expression of diabetes related genes and the stability of housekeeping gene expression can be considerably affected by metabolic dysregulation, *β*-cell malfunction, and dietary changes. It is technically not feasible to conduct quantitative analysis utilizing a single reference gene, as recommended by the Minimum Information for publication of Quantitative real-time PCR Experiments guidelines. To accurately evaluate gene expression profiles in diabetic mouse models, a set of suitable reference genes must express minimal variability in expression across different organs and tissues of interest, experimental samples, and conditions [19–21]. Several housekeeping genes have been widely used in mice investigations on obesity and diabetes studies [19,20,22,23]. However, there has been a lack of research that has examined the identification of a suitable set of reference genes for assessing the expression of diabetes related genes across several tissues, including the liver and pancreatic tissues.

In this study, we aim to systematically analyze the stability of 11 candidate reference genes, including *β-actin* (*ACT*), *β-2 microglobulin* (*B2M*), *Glyceraldehyde-3-phosphate dehydrogenase* (*GAPDH*), *Hypoxanthine phosphoribosyl transferase 1* (*HPRT1*), *Peptidylprolyl isomerase A* (*PPIA*), *Ribosomal protein L13A* (*RPL13A*), *Ribosomal protein subunit P0* (*RPLP0*), *TATA box binding protein* (*TBP*)*, Ubiquitin* (*UBC*), *Tyrosine 3-monoxygenase/tryptophan 5-monooxygenase activation protein – zeta polypeptide* (*YWHAZ*), and *18S ribosomal RNA* (*18SRNA*). The selection of the 11 candidate reference genes was based on their widespread use and reported stability in quantitative gene expression studies in obese and diabetic mice and rats across different tissues and cell types, including liver and pancreas [24–26]. Next, we aim to establish a reliable set of internal reference genes for accurate and reliable normalization of the gene expression in liver and pancreatic tissues across mice representing physiological (control), pathological (obese and diabetic), and treatment (treated STZ-diabetic) conditions. Evaluating potential candidate reference genes under the incorporation of curcumin exposure facilitates the evaluation of candidate reference genes under a relevant therapeutic scenario, thereby validating that the selected genes maintain stability across various physiological, pathological, and treatment conditions in diabetic mouse models. To ensure robust and reliable identification of the stable reference genes, four widely accepted statistical tools, including geNorm, NormFinder, BestKeeper, and the comparative delta Ct, were employed [27–30]. The data from these tools provide a rigorous framework to rank candidate genes and identify the most suitable reference genes for accurate normalization in RT-qPCR derived data.

## Materials and methods

### Animals and tissue samples

All animal experiments were reviewed and approved by the Institute of Genome Research – Institutional Review Board in Bio-Medical Research (Decision No.: 06/NCHG-HĐĐĐ; dated 20 September 2023). All procedures were performed in strict accordance with the guideline and all efforts were made to minimize animal suffering. The requirement for informed consent was waived, as the study involved only animal experiments.

Male Swiss albino mice (*Mus musculus* L. var. albino) weighing 20 ± 2 g were acquired from the National Institute of Hygiene and Epidemiology (NIHE). They were housed in plastic cages and maintained in a 12-hour-light/dark cycle with a humidity level of 60–70%. All types of diets and bedding material in this study were prepared and provided by the NIHE. A total of 48 mice were categorized into four distinct groups (n = 12 each): the control group (Group 1), the obesity group (Group 2), the diabetic group (Group 3), and the obesity, diabetic, and treated STZ-diabetic group (Group 4). Mice in Group 1 were fed with a normal-fat diet (NFD, Group 1, control group). Mice in Groups 2–4 were fed a high-fat diet (HFD), following the description by Nguyen *et al.* [31] prior to diabetic induction by streptozotocin (STZ, Sigma-Aldrich, USA). Mouse body weight was measured at 2-week intervals.

To establish a diabetic mouse model, mice in Groups 3–4 were injected intraperitoneally with 35 mg/kg body weight of STZ. Mice in Groups 1–2 were injected with 0.1 M citrate buffer. All mice were given access to their respective diets for 10–12 days and checked for blood glucose concentration by intraperitoneal glucose tolerance test (IPGTT) using a glucometer (Accu-Chek Instant, Roche, Switzerland) [32]. A blood glucose level over 11.1 mmol/L indicated effective establishment of a diabetic mouse model [6].

Diabetic mice from Group 4 were fed with a HFD diet supplemented with turmeric extract that is rich in the curcumin compound at an amount of 100 mg/kg body weight/day [33]. In contrast, Groups 1–3 were fed their respective diets. The experiment was conducted for 5 weeks. At the end of the experiment, the weight and blood glucose concentration of mice from all four groups were measured. Tissue samples (liver and pancreas) were collected and stored in RNA-later solution at -80^o^C after the mice were euthanized by cervical dislocation in accordance with the AVMA Guidelines for the Euthanasia of Animals 2020 [34]. In brief, a tweezer is placed behind the ears at the base of the skull of the mouse to stabilize the head, while firm pulling force is applied at the base of the tail to disarticulate the cervical vertebrae. This euthanasia method ensures rapid loss of consciousness, minimizes pain, and complies with ethical standards in research. All experiments were performed in triplicate.

### Total RNA extraction and complementary DNA synthesis

Total RNA extraction was performed on homogenized liver and pancreatic tissues using the RNeasy kit (Qiagen, Germany) following the manufacturer’s protocol. The assessment of RNA sample concentration and quality was conducted using a Nanodrop^TM^ spectrophotometer (Thermo Fisher, USA). High-quality RNA, indicated by an A_260_/A_280_ ratio ranging from 1.8 to 2.0, was utilized for the synthesis of cDNA. CDNA synthesis was performed on the Mastercycler Vapor Protect thermocycler (Eppendorf, Germany) using 1.0 μg of total RNA and the ProtoScript® II First Strand cDNA Synthesis Kit (NEB, Vietnam), following the manufacturer’s protocol.

### Quantitative real-time PCR analysis

The quantitative RT-qPCR analysis was conducted on a Rotor-Gene Q instrument (Qiagen, Germany) utilizing the Luna^®^ Universal One-Step RT-qPCR Kit (NEB, Vietnam). Each RT-qPCR reaction (20 µL), consisting of 1X Master mix, 1.0 mM of each primer, and 50 ng of cDNA sample, was amplified in one cycle at 95°C for 10 minutes and 40 cycles of 95°C for 15 seconds, then 60°C for 40 seconds. The candidate reference genes for normalization of RT-qPCR data derived from liver and pancreatic tissues of 4 experimental groups were *ACT*, *B2M*, *GAPDH*, *HPRT1*, *PPIA*, *RPL13A*, *RPLP0*, *TBP, UBC*, *YWHAZ*, and *18SRNA*. Our choice was guided by previous reference gene validation studies in obese and diabetic mouse/rats reported by Ai *et al.*, *Febriza et al*., and Secio-Silva *et al.* [24–26]. Their primers were designed using the NCBI Primer–BLAST (RRID: SCR_003095) tool (Table 1). The specificity of each pair of primers was analyzed by melting curves. The RT-qPCR data were evaluated with Livak’s ΔΔCt method [17]. All RT-qPCR analyses were performed in triplicate.

**Table 1:** List of candidate reference genes and target genes.

Primers	Accession number	Sequence (5’-3’)	Amplicon (bp)	Efficiency (%)
ACT	NM_007393.5	F: GTGTGACGTTGACATCCGTAAAG R: GCCGGACTCATCGTACTCC	247	103
B2M	NM_009735.3	F: GACCGGCCTGTATGCTATCC R: TTTCAATGTGAGGCGGGTGG	125	100
GAPDH	NM_001289726.2	F: CCCAGCAAGGACACTGAGCAAG R: CCCTCACAATTTCCATCCCAGACC	86	99
HPRT1	NM_013556.2	F: GGTTAAGCAGTACAGCCCCA R: TCCAACACTTCGAGAGGTCC	73	100
PPIA	NM_008907.2	F: GTGTTCTTCGACATCACGGC R: TAAAGTCACCACCCTGGCAC	187	100
RPL13A	NM_009438.5	F: CTGCCCCACAAGACCAAGAG R: GGACCACCATCCGCTTTTTC	100	97
RPLP0	NM_007475.5	F: CTGCACTCTCGCTTTCTGGA R: ACGCGCTTGTACCCATTGAT	113	102
TBP	NM_013684.3	F: GTGCCAGATACATTCCGCCT	121	97
		R: GCTGCGTTTTTGTGCAGAGT		
UBC	NM_019639.4	F: AGCCCAGTGTTACCACCAAGA R: TAAGACACCTCCCCCATCACA	117	99
YWHAZ	NM_001253807	F: GAAAAGTTCTTGATCCCCAATGC R: TGTGACTGGTCCACAATTCCTT	134	104
18SRNA	NR_003278.3	F: TTGACTCAACACGGGAAACC R: AGACAAATCGCTCCACCAAC	127	98
SLC2A2	NM_031197.2	F: TCCTTGGGCCTTACGTGTTC R: CTGGTCGGTTCCTCGGTTTT	230	99
GCK	NM_010292.5	F: CAACTGGACCAAGGGCTTCAA R: TGTGGCCACCGTGTCATTC	133	98

### Evaluation of the efficiency and stability of candidate reference genes

The efficiency of candidate reference genes was calculated by the qPCR Efficiency Calculator web-based tool (Thermo Fisher, USA) using the equation:


$$\text{PCR}\;\text{efficiency}=(\text{10}\;\;\hat{\;}\;([-\text{1}/\text{slope}])-\text{1})\times \text{100}$$

The slope for each candidate reference gene was generated by the RT-qPCR standard curve, which involved a serial dilution of 1:5, 1:25, 1: 50; 1:250 and 1:1250.

The stability of candidate reference genes was evaluated using three different algorithms, including geNorm (RRID: nlx_156922), NormFinder (RRID: SCR_003387) and BestKeeper (RRID: SCR_003380) and comparative ∆Ct methods [27,35,30]. The four popular methods, geNorm, NormFinder, BestKeeper, and comparative ΔCt, utilize different statistical methodologies for evaluating the stability of reference genes in RT-qPCR. GeNorm evaluates the stability of genes by analyzing pairwise variation, based on the assumption that the most stable genes display minimal expression levels. NormFinder utilizes a model-based approach that considers both intra- and inter-group variations to determine the most stable gene. BestKeeper employs raw Ct values to evaluate stability through standard deviation and correlation analysis, while the comparative ΔCt method assesses expression consistency by analyzing the geometric mean of the gene stability rankings derived from all methods.

The stability performance of the candidate reference genes was ranked according to their stability values (*M*), with the lowest *M* value considered the most stably expressed reference gene and *vice versa*. The optimal number of candidate reference genes for RT-qPCR analysis was determined by the pairwise variation (*V*_*n*_*/V*_*n+1*_) function of the geNorm tool. The proposed cut-off threshold value for pairwise variation analysis was 0.15, according to the recommendation of Vandesompele [27].

### Statistical analysis

Statistical analysis was conducted using Student’s t-test and one-way ANOVA (RRID: SCR_002427) with Tukey’s post hoc analysis [36]. The statistical significance (*p*-value ≤ 0.05) was denoted by distinct letters or numbers of asterisk symbols on each histogram.

## Results and discussion

### Body weight and blood glucose levels

**Fig 1:**
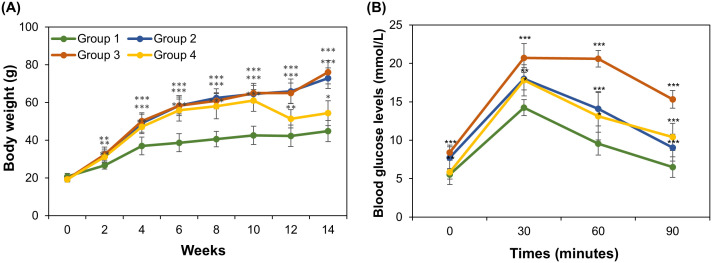
Changes in body weight (A) and blood glucose level (B) across four experimental groups of mice. **(A)** Body weight was measured at 2-week intervals. The period from week 0 to 8 is the development stage of obese mice (Groups 2-4). Post-diabetic induction by STZ, the diet of Group 4 was changed to HFD supplemented with curcumin powder; the diets of Groups 1-3 remained unchanged. **(B)** Blood glucose levels were measured at the end of the experiment (week 14). Statistical analysis was conducted by Student’s t-test to compare the weight and blood glucose level of Groups 2-4 with the control Group 1 with *p ≤ 0.05, **p ≤ 0.01, and ***p ≤ 0.001.

The body weight of mice in three experimental groups was increased (*p*-value < 0.001) in comparison to their counterparts in control Group 1 (Fig 1). In the first 10 weeks, the weight of mice in experimental Groups 2, 3, and 4, which were fed with the HFD diet, increased by 3.36 (from 19.2 to 64.5 g), 3.37 times (from 19.3 to 65.0 g), and 3.18 (from 19.2 to 61.0 g), respectively. It has been well established that diabetic mouse models can be developed by injecting induced obesity mice with a low dose of STZ [2,6]. Mice in Groups 3 and 4 were found to develop a diabetic condition 12 days post STZ injection, which was evidenced by the increased level of blood glucose 90 minutes after intraperitoneal injection of glucose solution, reaching 12.2 and 11.8 mmol/L, respectively. Post STZ injection at the end of week 10, there was a clear change in body weight of mice in Groups 2 and 3, which continued to feed with HFD, and those in Group 4, which fed with the HFD diet supplemented with curcumin powder. Turmeric extract powder has been found to significantly reduce body fat and restore insulin sensitivity and homeostasis in diabetic mice [12,25,37]. At the end of the experiment, as expected, the body weight of mice in Groups 2 and 3 continued to rise, reaching 72.9 and 76.0 g, respectively (Fig 1A). While the introduction of a new diet led to a reduction in the body weight of mice in Group 4; it decreased from 61.0 g to 54.3 g in just 4 weeks. On the other hand, the weight of mice in Group 1 has been found to rapidly increase in the first 10 weeks, reaching 42.6 g, before this trend was slowed down and reached approximately 44.8 g in week 14. [5].

The blood glucose levels of mice from all four groups were also measured at week 14. Fig 1B reveals that the glucose level was increased to the highest level of 14.2, 18.0, 20.7 and 17.8 mmol/L in all 4 groups, respectively, after 30 minutes of glucose injection. This trend was gradually dropped after 90 minutes (Fig 1B). Specifically, blood glucose levels of Groups 1, 2, and 4 were below the diabetic threshold of 11.1 mmol/L, such as 6.5, 9.0, and 10.9 mmol/L, respectively. In contrast, the blood glucose level of mice in Group 3 remained at a high level of more than 15.4 mmol/L after 90 minutes of glucose injection [6].

### Expression profiles of candidate reference genes in liver and pancreatic tissues

Prior to the expression profile of eleven candidate genes in liver, pancreatic, and across both types of tissues, the efficiency of their primers was assessed by RT-qPCR standard curves and the qPCR efficiency calculator web-based tool (Table 1). The obtained results reveal that the efficiency of eleven sets of primers varied from 97 to 104% (Table 1, S1 Fig. and S1 File). Among them, five set primers, such as *B2M*, *GAPDH*, *HPRT1*, *PPIA,* and *UBC*, obtained an efficiency of 99–100%, indicating that the primers and polymerase enzyme are working at their maximum capacity, amplifying the target sequence efficiently. However, it is also widely acceptable to obtain the RT-qPCR primer efficiency in a range of 90–110% [38,39]. Therefore, the primer sets of the remaining six genes are considered ideal for the RT-qPCR assessment.

**Fig 2:**
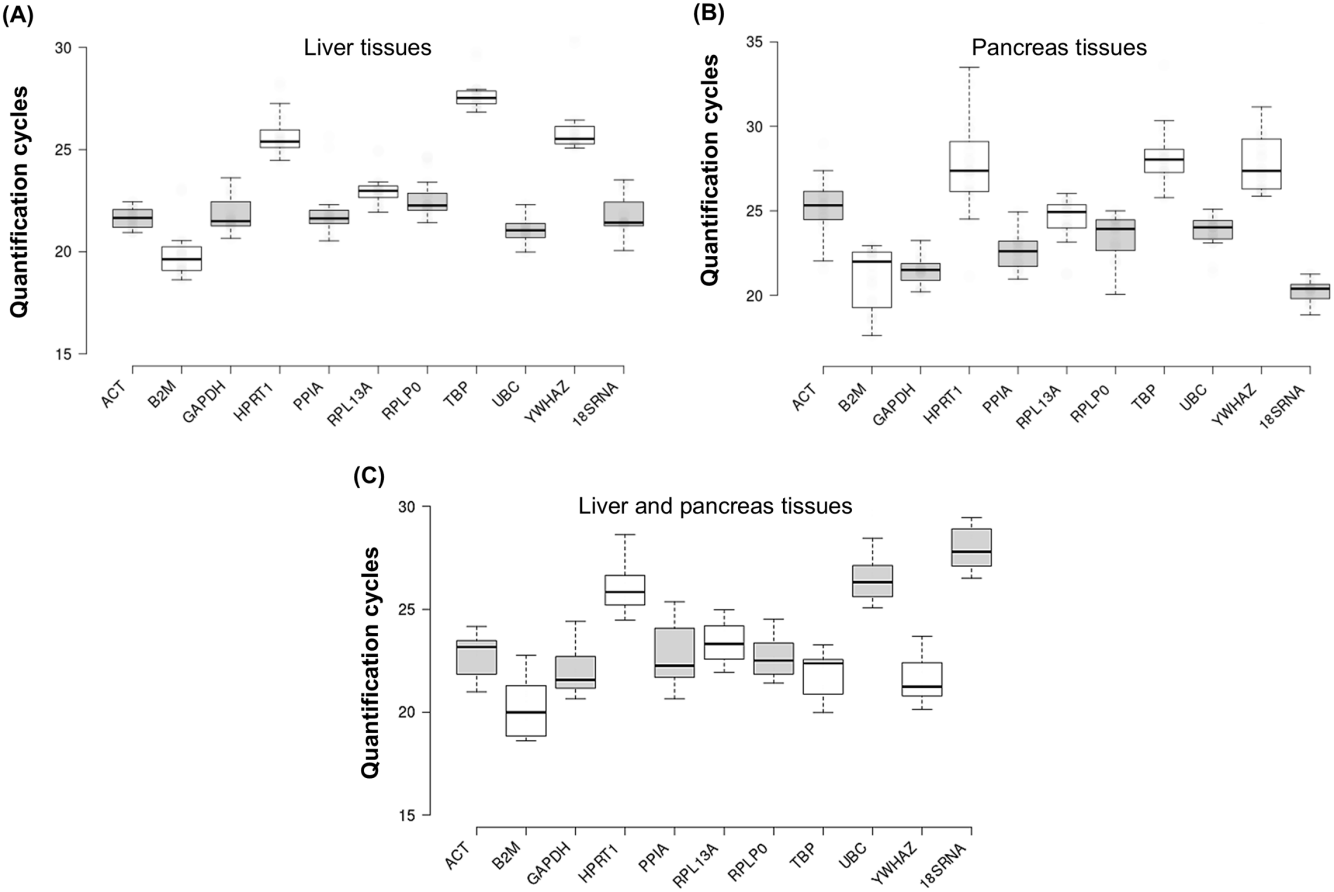
Distribution of quantification cycles (Cq) for reference genes in liver (A), pancreas (B), and combined liver-pancreas tissues (C). The assessed reference genes yielded an average Cq value that varied from 19.6 to 27.5, 20.4 to 28.1, and 20.0 to 27.8 in the liver, pancreas, and both types of tissues, respectively. The whisker caps illustrate the upper and lower distributions of the Cq values, whereas the boxes signify the first and third quartiles. The line within the box represents the median value.

Expression profiles of eleven candidate reference genes were evaluated in four experimental groups of mice across liver, pancreas, and combined liver-pancreas tissues. Fig 2 reveals that the average Cq values for eleven candidate reference genes varied from 19.6 to 27.5 and 20.4 to 28.1 in liver and pancreatic tissues, respectively, with the lower Cq value indicating a more abundant target transcript. Fig 2A and 2B reveal that the majority of candidate genes have the average Cq value in a range of 21.5 to 23.0 and 20.4 to 24.9 in the liver and pancreatic tissues, respectively. Among them, *RPL13A*, *PPIA*, *UBC*, and *ACT* have the least variance in the distribution of the Cq values in liver tissues (Fig 2A). In contrast, *GAPDH* and *18SRNA* had the widest range of Cq values. In pancreatic tissues, the most compact Cq value distribution was found in *18SRNA*, *UBC,* and *GAPDH*, and the least compact Cq distribution was observed in *B2M*, *HPRT1*, and *YWHAZ* (Fig 2B). When liver and pancreas tissues were evaluated together, the candidate reference genes mainly returned the average Cq values within the range of 20.0 to 23.3. The least variance in the distribution of the Cq values was found in *RPL13A*, *RPLP0*, and *UBC* (Fig 2C). These results indicated that genes, which have the least variance in the distribution of the Cq values, are considered the most stably expressed candidate reference genes for accurate normalization of RT-qPCR derived data from liver, pancreatic, and combined liver-pancreas tissues across four experimental groups.

### Expression stability of candidate reference genes

The stability of eleven candidate reference genes was evaluated by four popularly used tools, geNorm, NormFinder, BestKeeper, and comparative ∆Ct, aimed at evaluating and selecting reference genes from experimental datasets (Table 2). The candidate reference genes were evaluated based on their stability (*M*) values derived from stepwise exclusion, alongside the variability observed in intra- and inter-group analyses using geNorm and NormFinder algorithms. Additionally, the coefficient of variation for reference genes was determined through the BestKeeper algorithm, and the standard deviation of the Cq values was calculated using the comparative ∆Ct method across all tissue samples assessed, including liver, pancreas, and both types of tissues. Considering that these tools evaluate the stability of potential reference genes using various methodologies [27–30], it is anticipated that there will be variation in the stability ranking of eleven candidate reference genes generated by distinct statistical algorithms across all examined tissues.

**Table 2:** Assessment of the stability of eleven candidate reference genes across liver tissues of 4 experimental groups of mice by the geNorm, NormFinder, BestKeeper, and comparative ∆Ct methods.

Tissues	GeNorm		NormFinder		BestKeeper		Comparative ∆Ct		Consensus	
	Gene	*M* value*	Gene	*M* value*	Gene	*r* value**	Gene	Mean SD***	Gene	Geometric mean of ranking
Liver	*ACT*	0.48	*ACT*	0.17	*PPIA*	0.99	*RPL13A*	0.46	*ACT*	1.86
	*RPL13A*	0.49	*TBP*	0.19	*B2M*	0.97	*RPLP0*	0.46	*RPL13A*	2.21
	*TBP*	0.50	*RPL13A*	0.20	*ACT*	0.97	*TBP*	0.48	*TBP*	3.35
	*GAPDH*	0.52	*HPRT1*	0.21	*RPL13A*	0.95	*ACT*	0.48	*PPIA*	3.81
	*PPIA*	0.54	*UBC*	0.22	*UBC*	0.95	*UBC*	0.50	*UBC*	5.18
	*HPRT1*	0.56	*PPIA*	0.24	*HPRT1*	0.94	*GAPDH*	0.51	*HPRT1*	5.79
	*UBC*	0.57	*GAPDH*	0.24	*YWHAZ*	0.93	*HPRT1*	0.53	*B2M*	5.98
	*B2M*	0.58	*B2M*	0.27	*TBP*	0.92	*PPIA*	0.57	*GAPDH*	7.01
	*YWHAZ*	0.63	*YWHAZ*	0.30	*GADPH*	0.84	*18SRNA*	0.59	*RPLP0*	7.49
	*18SRNA*	0.63	*RPLP0*	0.32	*18SRNA*	0.81	*B2M*	0.59	*YWHAZ*	8.55
	*RPLP0*	0.71	*18SRNA*	0.32	*RPLP0*	0.80	*YWHAZ*	0.76	*18SRNA*	9.43
Pancreas	*UBC*	1.02	*UBC*	0.16	*UBC*	0.96	*UBC*	1.22	*UBC*	1.00
	*18SRNA*	1.03	*18SRNA*	0.18	*RPL13A*	0.84	*RPL13A*	1.27	*RPL13A*	2.83
	*TBP*	1.14	*RPL13A*	0.22	*RPLP0*	0.81	*RPLP0*	1.28	*18SRNA*	3.31
	*RPL13A*	1.18	*PPIA*	0.25	*18SRNA*	0.74	*18SRNA*	1.29	*RPLP0*	3.81
	*RPLP0*	1.22	*TBP*	0.27	*ACT*	0.71	*PPIA*	1.36	*TBP*	4.90
	*PPIA*	1.30	*GADPH*	0.30	*YWHAZ*	0.67	*GAPDH*	1.42	*PPIA*	5.73
	*GAPDH*	1.31	*RPLP0*	0.32	*HPRT1*	0.66	*ACT*	1.56	*GAPDH*	7.26
	*YWHAZ*	1.41	*B2M*	0.34	*TBP*	0.65	*TBP*	1.69	*ACT*	7.69
	*B2M*	1.48	*YWHAZ*	0.40	*PPIA*	0.54	*B2M*	1.82	*YWHAZ*	8.30
	*ACT*	1.49	*ACT*	0.51	*B2M*	0.45	*HPRT1*	2.13	*B2M*	8.97
	*HPRT1*	1.50	*HPRT1*	0.73	*GADPH*	0.40	*YWHAZ*	2.14	*HPRT1*	9.59
Liver & pancreas	*PPIA*	0.96	*TBP*	0.09	*RPL13A*	0.89	*RPL13A*	0.86	*RPL13A*	2.21
	*TBP*	0.98	*UBC*	0.17	*ACT*	0.86	*RPLP0*	0.87	*UBC*	3.22
	*RPL13A*	1.03	*RPLP0*	0.18	*UBC*	0.84	*UBC*	0.90	*TBP*	3.46
	*YWHAZ*	1.07	*GAPDH*	0.21	*YWHAZ*	0.81	*18SRNA*	0.93	*RPLP0*	3.81
	*RPLP0*	1.09	*YWHAZ*	0.22	*HPRT1*	0.79	*PPIA*	0.95	*PPIA*	4.05
	*UBC*	1.11	*B2M*	0.23	*PPIA*	0.77	*GAPDH*	0.96	*YWHAZ*	5.45
	*GADPH*	1.12	*18SRNA*	0.23	*RPLP0*	0.75	*ACT*	1.02	*ACT*	6.26
	*B2M*	1.15	*RPL13A*	0.23	*B2M*	0.67	*TBP*	1.09	*GADPH*	6.56
	*HPRT1*	1.33	*PPIA*	0.26	*TBP*	0.65	*B2M*	1.20	*18SRNA*	7.27
	*18SRNA*	1.46	*ACT*	0.34	*18SRNA*	0.54	*HPRT1*	1.32	*B2M*	7.67
	*ACT*	1.50	*HPRT1*	0.49	*GADPH*	0.44	*YWHAZ*	1.45	*HPRT1*	8.39

* M values: stability values evaluated by the geNorm and NormFinder algorithms with the smaller M value indicating the more stable the gene expressed in tissues assessed; ** r values: Pearson correlation coefficient values evaluated by the BestKeeper algorithm with the higher r value indicating the better reference gene; *** mean SD: mean standard deviation value calculated by the comparative ∆Ct method with the smaller SD value indicating the better choice for selection of stably expressed reference genes across evaluated tissue samples.

For liver tissues, the geNorm algorithm has determined that *ACT* > *RPL13A* > *TBP* are the most stable reference genes, exhibiting the smallest M values in that order. While *RPLP0* exhibited the highest level of instability among the reference genes analyzed. NormFinder identified *ACT* > *TBP* > *RPL13A* as the most stable gene pair, while *18SRNA* was found to be the least stable pair for normalization of RT-qPCR derived data. The BestKeeper tool ranked *PPIA* > *B2M* > *ACT*, identifying them as the most appropriate for RT-qPCR normalization due to their high coefficient of variation, while *RPLP0* was ranked the lowest. Through the analysis of the standard deviation of Cq values, the comparative ∆Ct method selected *RPL13A > RPLP0* > *TBP* as the optimal combination for accurate and reliable normalization of RT-qPCR data, while *YWHAZ* was identified as the worst one.

In pancreatic tissues, the analysis conducted using the geNorm and NormFinder algorithms revealed that *UBC* > *18SRNA* > *TBP* represents the most stable reference genes for normalization of RT-qPCR data. In contrast, *HPRT1* was identified as the least stable reference gene with the lowest stability by both tools employed in the analysis. The study conducted using the BestKeeper and comparative ∆Ct method indicated that the genes exhibiting the highest rankings were *UBC* > *RPL13A *>* RPLP0*. Whereas those with the lowest rankings were *GAPDH* and *YWHAZ*, respectively. It is important to emphasize that the variation noted among the four statistical approaches employed to evaluate the stability of eleven reference genes was negligible. Within this particular tissue, *UBC* consistently attained the highest ranking across all analytical tools. Furthermore, *18SRNA*, *RPLP0*, and *RPL13A* exhibited high rankings, indicating a robust group of candidates for accurate RT-qPCR normalization.

In the analysis of liver and pancreatic tissues, based on geNorm *M* values, the candidate reference genes exhibiting the greatest stability were *PPIA* > *TBP *> *RPL13A*. Analysis using NormFinder identified that the best reference genes were *TBP* > *UBC *> *RPLP0*. BestKeeper ranked *RPL13A* > *ACT* > *UBC* as the most appropriate candidates for normalization of RT-qPCR data. The comparative ∆Ct method revealed that the reference genes with the highest stability of expression were *RPL13A* > *RPLP0 *> *UBC*. On the other hand, the candidate reference genes identified as the least stable were *ACT, HRPT1, GAPDH,* and *YWHAZ,* as determined through the analyses conducted using geNorm, NormFinder, BestKeeper, and comparative ∆Ct methods, respectively.

To minimize the discrepancies among the four statistical tools in the liver and across both liver and pancreatic tissues, it is recommended to evaluate the comprehensive ranking of reference genes by calculating the geometric mean of ranking from all the assessed statistical algorithms [27,40]. The geometric mean (GM) of each candidate gene was computed to determine its integrated ranking, with a lower value signifying greater expression stability [41]. The reference genes identified with the lowest GM ranking demonstrate the highest suitability for ensuring reliable and accurate normalization of RT-qPCR data. The geometric mean analysis reveals that *RPL13A* stands out as the most stable reference gene among all examined tissues and under different experimental conditions, including control, obese, diabetic, and treated STZ-diabetic mice. This is supported by the GM values recorded at 2.21, 2.83, and 2.21 for the liver, pancreas, and both types of tissues, respectively (Table 2). *RPL13A* functions as a housekeeping gene that encodes the ribosomal protein L13A subunit. RPL13A is a member of the L13P family of ribosomal proteins, which are crucial components for cell development and tissue homeostasis [24]. *RPL13A* has been frequently used as a reference gene for many studies due to consistent expression across various tissue samples (liver, pancreas, adipose, cortical bone) and experimental conditions, including those associated with obesity and diabetes [19,24,42–44].

According to the values of GM of ranking, the other top-ranked reference genes for liver were *ACT* with a GM value of 1.86 (Table 2). In previous studies, *ACT* has severed as a reference gene for normalization of RT-qPCR data derived from liver tissues of mouse models suffering from conditions of obesity, diabetes, and liver diseases. In 2016, Gong and his colleagues determined that *ACT* ranks as one of the most stably expressed genes (ranked 2 out of 12 genes), making it an optimal choice for a reference gene to assess the abundance of mRNA transcripts in the liver tissues of male C57BL/6 mice [40]. Similarly, *ACT* has been utilized to normalize the expression levels of various genes in mice subjected to HFD-fed and those that are genetically mutated (*ob/ob*) mice [45].

The consensus results of the four statistical tools assessed also identified that *UBC*, together with *RPL13A*, are the most stably expressed candidate reference genes for normalization of RT-qPCR data derived from pancreas and from both pancreas and liver tissues. The GM values of *UBC* were recorded as 1.0 and 3.22 in pancreatic tissues as well as in both types of tissues, respectively (Table 2). This pair of genes has served as reference genes for the normalization of RT-qPCR data obtained from pancreatic *β*-cells of the diabetic C3HeB/FeJ inbred mouse strain [46]. The *UBC* has also been evaluated as a stable expressed reference gene across liver cell types in mouse and rat models [47,48].

The genes *GAPDH*, *18SRNA*, *YWHAZ*, and *HPRT1* have been utilized either as a singular reference gene or as components of an optimal set for the normalization of qPCR data [49,50]. Our findings indicated that these genes were among the inappropriate candidate reference genes in the liver and pancreatic tissues. In alignment with our findings, previous studies identified *YWHAZ* [40] as the least suitable reference gene for gene expression profiling of liver, muscle, and adipose tissues of HFD-fed obese mouse models, whereas *B2M* was noted as the most unstable reference gene for liver, pancreas, and kidney [26]. In a similar manner, *HPRT1*, *GAPDH*, and *18SRNA* have been identified as having the lowest geNorm M values in liver tissues of C57BL/6 mice [20].

**Fig 3:**
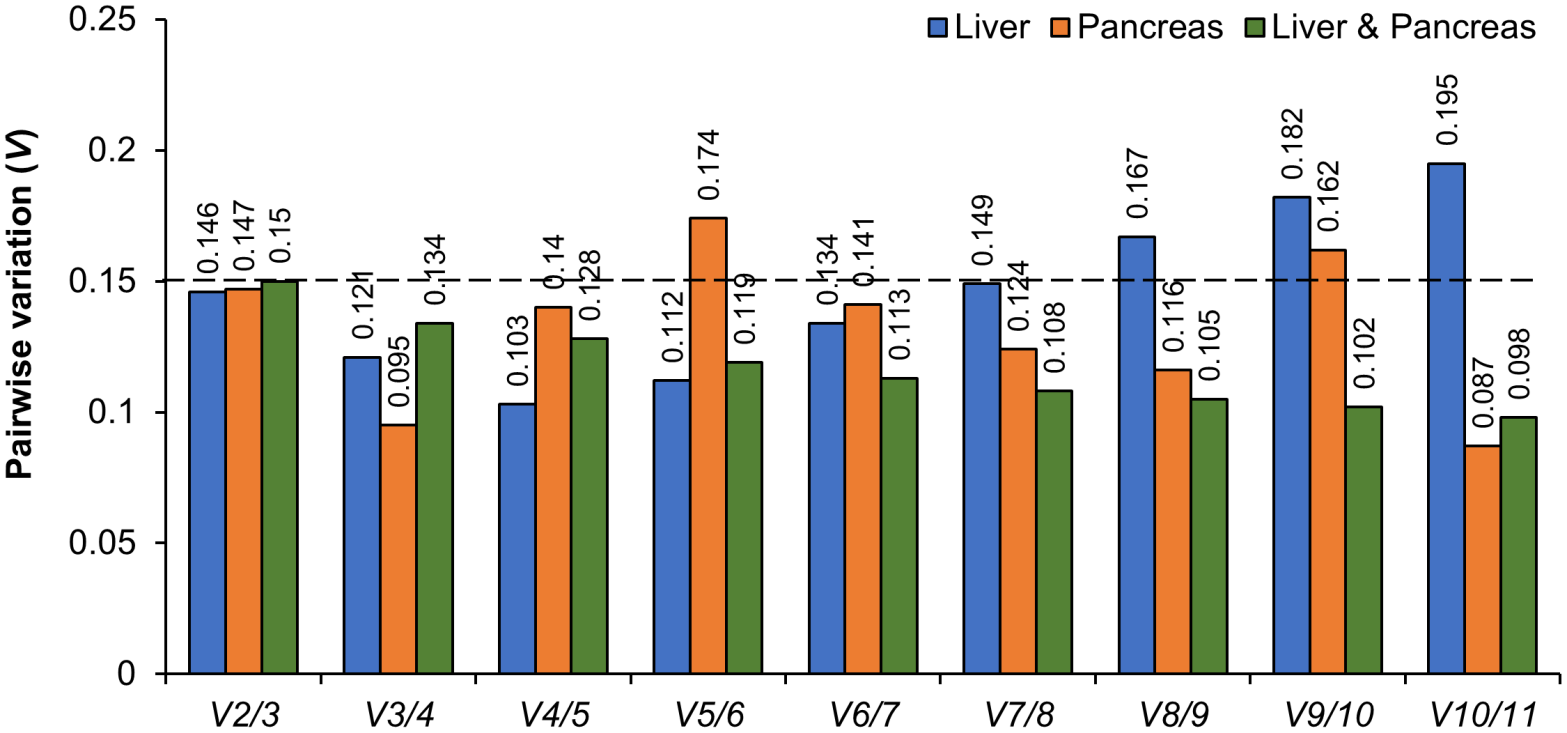
Pairwise variation (*V*_*n*_*/V*_*n*+*1*_) analysis of eleven candidate genes by geNorm for determining the optimal number of reference genes required for the accurate normalization of RT-qPCR derived data from liver, pancreas, and both types of tissues. The cut-off threshold was set at 0.15.

In the process of normalizing RT-qPCR data, it is possible to utilize a single reference gene in certain cases; however, this approach may lead to significant inaccuracies and biased outcomes. Consequently, it is advisable to utilize at least two reference genes to improve the precision and dependability of the normalization procedure [51,52]. The analysis of pairwise variation (*Vn/Vn + 1*) through geNorm has demonstrated its efficacy in identifying the optimal number of reference genes. Fig 3 reveals that the pairwise variation analysis yielded the *V*_*2/3*_ values of 0.146, 0.147, and 0.15 for liver, pancreas, and both types of tissues, respectively. The results obtained are below the recommended threshold of 0.15 [27]. Moreover, the inclusion of reference genes has shown a significant reduction in *V* values, particularly *V*_*3/4*_ (liver: 0.121, pancreas: 0.095 and both types of tissues: 0.134) and *V*_*4/5*_ (liver: 0.103, pancreas: 0.14 and both types of tissues: 0.128). Nevertheless, the optimal number of reference genes identified was two, as this was regarded as the most practical option. The present finding aligns with the research conducted by Vandesompele [27], suggesting that adding more reference genes does not substantially improve the robustness of gene expression evaluation.

### Validation of selected candidate reference genes

To assess the suitability of candidate reference genes for RT-qPCR normalization, both the most and least stable pairs were used to normalize the expression levels of *SLC2A2* (*Solute carrier family 2 member 2*) and *GCK* (*Glucokinase*) in liver and pancreatic tissues across the four experimental groups. *SLC2A2* is responsible for encoding glucose transporter 2 (GLUT2), which is predominantly expressed in the liver, pancreatic β-cells, intestine, and kidney [53]. *GCK* encodes a key enzyme involved in glucose metabolism, catalyzing the phosphorylation of glucose to form glucose-6-phosphate [54]. *SLC2A2* and *GCK* collaborate to regulate glucose sensing, uptake, and overall glucose homeostasis within the body, making them suitable targets for validating the stability of reference genes in liver and pancreas tissues [53,54].

**Fig 4:**
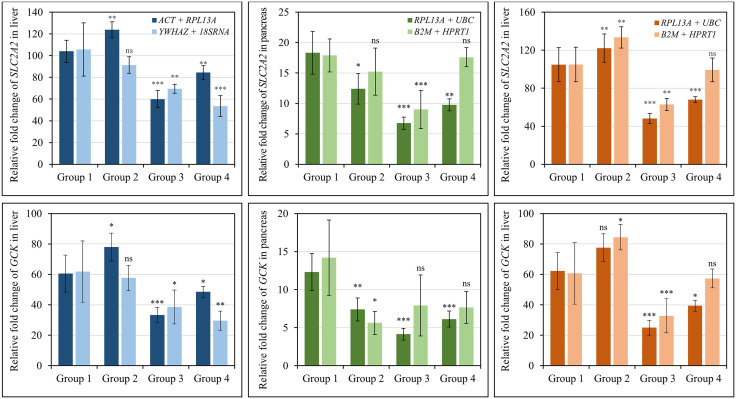
Relative fold changes of *SLC2A2* and *GCK* expression in liver and pancreas tissues across four experimental groups (1-4), normalized using the most stable (*ACT* + *RPL13A; RPL13A *+ *UBC*) and least stable (*YWHAZ *+ *18SRNA*; *B2M* *+ HPRT1*) reference gene combinations. Asterisks above the bars denote significant differences between experiment groups (2-4) and control group 1 (*p*-values: * ≤ 0.05; ** ≤ 0.005; *** ≤ 0.001). ns: not significant (*p*-value > 0.05).

In the liver, the use of the most stable pair (*ACT* + *RPL13A*) resulted in consistent expression patterns of *SLC2A2* and *GCK*. Particularly, the expression levels of *SLC2A2* and *GCK* exhibited an increase in Group 2, showing a 1.2-fold (*p*-value: 0.004) and 1.3-fold (*p*-value: 0.018) rise, respectively. In contrast, these levels were relatively reduced in Group 4 with reductions of 1.2-fold (*p*-value = 0.003 and 0.044, respectively) and greatly dropped in Group 3 by 1.7-fold and 1.8-fold (*p*-value < 0.001), respectively (Fig 4). The rise observed in the expression of two genes in the obese mice of Group 2 suggests an adaptive upregulation in response to hyperglycemia and metabolic changes commonly associated with obesity [55,56]. Whereas, in treated STZ-diabetic Group 4, the expression levels of *SLC2A2* and *GCK*, normalized against the most stable reference gene combination (*RPL13A* + *ACT*), showed a substantial reduction compared to the control (*p*-value < 0.01); however, this decrease was not as substantial as that in diabetic Group 3. In contrast, when normalized using the least stable reference genes (*YWHAZ* + *18SRNA*), the expression of *SLC2A2* and *GCK* in the liver showed no significant difference between Groups 1 and 2. However, the Group 4 exhibited relatively lower expression levels (*p*-values = 0.007 and 0.07) compared to the diabetic Group 3 (Fig 4).

In pancreas tissue, the expression of *SLC2A2* and *GCK* followed a similar pattern to that in liver tissue (Fig 4). Groups 2, 3, and 4 all showed a consistent decrease in *SLC2A2* and *GCK* expression levels when normalized using *RPL13A* + *UBC*, the most stable reference gene combination, in comparison to the control Group 1 [54]. In Group 3, the diabetic mice showed the most noticeable decrease, with reductions of 2.7- and 3.0-fold (*p*-values < 0.001), respectively. In contrast, the expression levels of diabetic mice in Group 4 were greater than those in Group 3 (*p*-value = 0.001 and 0.007, respectively), although their levels still comparatively lower (*p*-value > 0.05) than the levels observed in Group 2 (Fig 4). Different patterns of *SLC2A2* and *GCK* expression were seen when normalized to the least stable reference gene combination (*B2M* + *HPRT1*). In the mice from Groups 2 and 4, the expression level of *SLC2A2* did not differ from that of Group 1 (*p*-values > 0.05). Conversely, in Group 3, a 2.0-fold decrease (p-value = 0.001) was recorded. The expression of *GCK* was decreased to a greater level of 2.5-fold in Group 2 (*p*-value = 0.03) when compared to its normalization using the stable reference gene pair (1.7-fold, *p*-value = 0.002). Nonetheless, the expression of *GCK* showed no significant changes in Groups 3 and 4 (*p*-values > 0.05).

Furthermore, the *RPL13A* + *UBC* combination has also been identified as an appropriate set of reference genes for accurate and reliable normalization of both liver and pancreas tissues, as confirmed by all four statistical algorithms employed (Table 2). As expected, the expression profiles of *SLC2A2* and *GCK*, when normalized with this reference gene combination, exhibited a pattern similar to that observed using the stable reference genes (*RPL13A* + *ACT*) identified specifically for liver (Fig 4). The process of normalization using the least stable reference genes (*B2M* + *HPRT1*) resulted in comparable overall patterns; however, the data indicated slightly elevated expression levels compared to the stable pairs, highlighting the influence of reference gene selection on quantitative results.

This study has several limitations that should be acknowledged. Firstly, we did not conduct a correlation analysis between the reference genes and the characteristics of organs and pathological features, thereby preventing the evaluation of any potential associations. Furthermore, the investigation focused only on 11 reference genes based on previous literature. In future research, RNA-seq data will be essential for the identification of additional novel markers.

## Conclusion

In conclusion, this study provides a list of novel reference genes for the accurate and reliable normalization of RT-qPCR generated data derived from liver, pancreas, and both types of tissues across physiological, pathological, and treatment conditions. To the best of our knowledge, this work represents one of the first evaluations of reference gene stability for both liver and pancreas across four distinct experimental mouse groups. The reference genes identified as most stable for liver and pancreatic tissues were *RPL13A* and *UBC*. Our statistical analysis also identified two top-ranked gene pairs for RT-qPCR normalization, such as *ACT* and *RPL13A* for liver tissues and *RPL13A* and *UBC* for pancreatic tissues. The findings in this study offer valuable insights that assists the future profiling of genes of interest in mouse models affected by obesity, diabetes and treatment conditions. These results provide a reliable and robust framework for accurate normalization of RT-qPCR data in mouse liver and pancreas as well as highlight the necessity of validating reference gene stability under physiological, pathological, and treatment conditions.

## Supporting information

S1 Fig Amplification efficiencies of 11 candidate reference genes and two target genes (*SLC2A2*, *GCK*) generated by Illumina Q-Rex software for RT-qPCR normalization analysis. (DOCX)

S1 File Raw data. (XLSX)
