# Supplementary material for: Identification of stable internal reference genes for expression analysis in the liver and pancreas of diabetic mouse (Mus musculus L.) models under physiological, pathological and treatment conditions
Source: PLoS One. 2026 Jan 7;21(1):e0338403. doi: 10.1371/journal.pone.0338403 (PMC12779057; doi:10.1371/journal.pone.0338403)
Supplement: S1 Fig — (DOCX) [file pone.0338403.s001.docx]

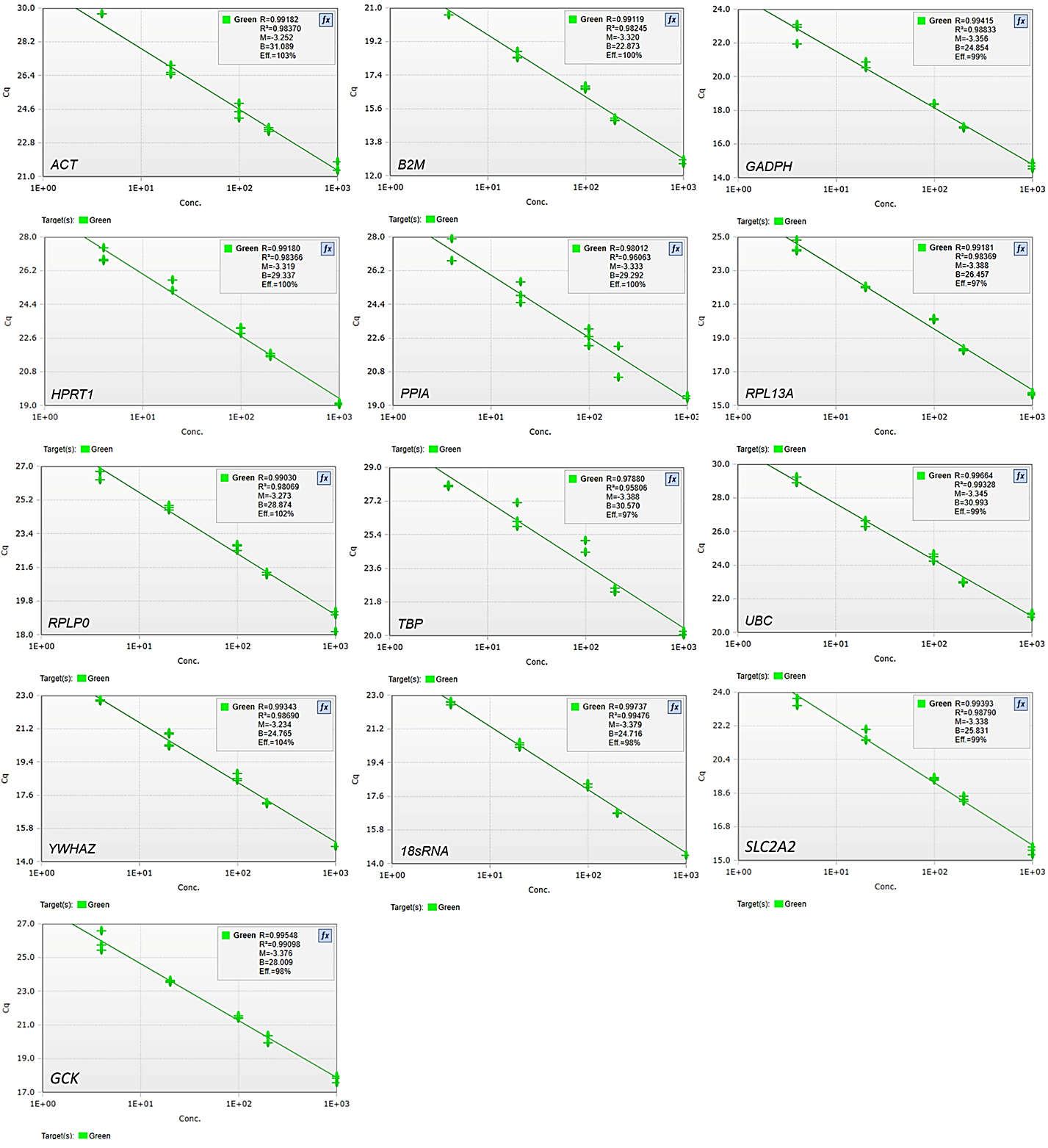


**Figure S1**. Amplification efficiencies of 11 candidate reference genes and two target genes (*SLC2A2*, *GCK*) generated by Illumina Q-Rex software for RT-qPCR normalization analysis. All primer sets utilized in this study demonstrated high specificity and exhibited high efficiency values, ranging from 97% to 104%.
